# Evolutionary dynamics of value co-creation within the industry-university-research network: A multi-agent game perspective

**DOI:** 10.1371/journal.pone.0338379

**Published:** 2025-12-04

**Authors:** Xuemin Zhang, Haijun Wang, Sardar M. N. Islam, Xiangfei Meng

**Affiliations:** 1 School of Management, Shenyang University of Technology, Shenyang, Liaoning, China; 2 Liaoning Administrators College of Police and Justice, Shenyang, Liaoning, China; 3 ISILC, Victoria University, Melbourne, Australia; University of Naples Federico II: Universita degli Studi di Napoli Federico II, ITALY

## Abstract

This paper investigates the evolutionary dynamics of value co-creation and defines the roles of multiple agents within the industry-university-research (IUR) network. A distinctive evolutionary game model from a multi-agent perspective is proposed to capture the previously under-explored dynamics of value co-creation within the IUR network. Furthermore, China’s high-end manufacturing industry was selected to establish the IUR network for simulation analysis because of its rapid transition toward innovation-driven value co-creation and the availability of granular data on IUR partnerships. The evolutionary game analysis yields three key findings with specific implications: Firstly, it reveals that the network’s average positive co-creation probability is inversely correlated with the enterprise investment difference and the academic knowledge input disparity between positive and negative value co-creation. This inverse relationship, identified by the game model, underscores the critical need for an effective supervision and management mechanism to optimize the utilization of enterprise investment and academic knowledge input during the value co-creation process within the IUR network. Secondly, the evolutionary game analysis demonstrates that specific parameters within the model – namely, the enterprise’s knowledge absorption capacity, the academic institution’s R&D capability, the government’s subsidy and penalty, and the intermediary’s transformation coefficient – play crucial roles in enhancing value co-creation probability. Based on these model-driven insights, we conclude that a combination of government guidance and market mechanisms serves as a sustainable driver for promoting value co-creation within the network. Thirdly, the model establishes an inverted U-shape relationship between the enterprise’s benefit distribution coefficient and the average positive co-creation probability. This critical finding from the evolutionary game simulation highlights the advantage of establishing an impartial interest distribution mechanism between industry and academia to foster synergy and stability in value co-creation within the IUR network.

## Introduction

The paradigm of technological innovation has gradually evolved from linear to nonlinear [1], and the network paradigm has been widely utilized to comprehend the Industry-University-Research (IUR) innovation. The IUR network, as a vital component of the national technological system, comprises multiple agents engaged in technological innovation and value creation [2]. Enterprises and academic institutions (universities and research institutes) collaborate in value co-creation through resource sharing and leveraging complementary advantages. At the same time, the government provides policy support and financial subsidies, and science and technology intermediaries provide service support [3]. In such a way, multiple entities cooperate to facilitate technological innovation and achievement transformation. Therefore, the IUR network provides strong support for optimizing the allocation of scientific and technological resources and improving the conversion rate of scientific achievements.

Nevertheless, due to differing goals and interests, cooperation between industry and academia may deviate from the intended path, leading to inefficiencies in value creation and the wastage of scientific and technological resources [4]. Therefore, understanding the IUR network and the value co-creation behaviors of multiple agents is crucial for enhancing the efficiency of value co-creation and ensuring the rational allocation of resources.

Existing research on the IUR network primarily focuses on network structures, examining indicators such as degree centrality, clustering coefficient, and structural holes from the perspective of the triple helix model [5,6]. While industry, academia, and government are key stakeholders, science and technology intermediaries also play significant roles in facilitating supply-demand matching and information flow within the network. However, considerably less attention has been paid to understanding the IUR network through a comprehensive multi-agent lens [7,8]. Furthermore, a critical gap remains regarding value co-creation dynamics: although interactions among agents have evolved from knowledge transfer to value co-creation [9], existing studies lack a thorough investigation into the evolutionary dynamics of value co-creation behavior within the multi-agent IUR system and the systemic factors influencing these dynamics, particularly those with strong practical relevance for industry [10]. The gap—specifically, the absence of a multi-agent evolutionary perspective to model and analyze the dynamics and drivers of value co-creation in the IUR network—is the focus of this research.

To bridge the gap in understanding the evolutionary dynamics of multi-agent value co-creation, this study employs evolutionary game analysis to investigate the mechanisms underlying this behavior within the IUR network. Specifically, it examines key indicators and strategic interactions from a multi-agent perspective with the evolutionary game analysis, a methodology which can uniquely capture how bounded-rational agents dynamically adapt their value co-creation strategies through learning and imitation over time. To ensure practical implications, the study constructs the IUR network of China’s high-end equipment manufacturing industry using patent data and conducts simulations to analyze agent co-creation behavior. Precisely, the research seeks to address the following inquiries: (1) What factors influence value co-creation in the IUR network from a multi-agent game perspective? (2) How do these factors impact the evolution of value co-creation within the network? (3) What roles do multiple agents play in value co-creation within the IUR network?

This paper aims to contribute in several ways: Firstly, it deconstructs key influencing factors through a multi-agent evolutionary game model, revealing how enterprise knowledge absorption, academic R&D capabilities, government policies, and intermediary roles collectively shape value co-creation dynamics. Secondly, it maps the evolutionary trajectory by thoroughly examining how agent interactions and strategy co-evolution impact the system’s convergence toward stable co-creation states. Thirdly, it clarifies agent roles and validates findings by simulating strategy evolution within China’s empirical high-end equipment manufacturing IUR network, moving beyond virtual network studies to demonstrate practical synergy mechanisms.

The remainder of the paper is structured as follows: Section 2 provides a theoretical background on the IUR network and value co-creation, establishing an analytical framework. Section 3 outlines the model assumptions and presents the network evolutionary game model. Section 4 details the construction of the IUR network within China’s high-end equipment manufacturing industry. Section 5 offers insights from simulation runs on value co-creation from a multi-agent game perspective. Finally, Section 6 concludes the study and suggests potential avenues for future research.

## Literature review and research framework

### Industry-university-research network

Industry-university-research network refers to the network system formed by enterprises, universities, and research institutes to expand resources and capabilities and carry out technological innovation cooperation [11]. Previous research has emphasized the significance of the IUR network and analyzed the network structure and position from the perspective of diverse network agents [1,12]. As valuable agents in IUR cooperation, enterprises, academic institutions (universities and research institutes), governments, and technology intermediaries have been discussed respectively. Enterprises have become the leading part of the IUR network by striving to break through the limitations of their own resource endowments, seek complementary technologies, and obtain lasting competitive advantages [1]. However, there are significant differences in the knowledge absorption capacity of various enterprises [13], which impacts knowledge transfer in the IUR cooperation [14].

Academic institutions, which provide innovative support for enterprises [15], are considered the primary sources of knowledge and technology in the IUR network. The research and development (R&D) capability, technology transfer capacity, and reputation of universities are positively correlated with their network centrality, which, in turn, fosters open innovation within the cooperation network [11,16]. Further research shows that there is a linear or nonlinear relationship between the network centrality of research institutes and their innovation performance in the IUR network with varying degrees of heterogeneity [6].

The involvement of the government in Industry-University-Research (IUR) networks has been extensively examined in academic literature, especially concerning the triple helix theory [17,18]. Government policies directly affect the performance of cooperative innovation [19]. In order to promote IUR cooperation, the government provides policy and financial support for cooperation projects between industry and academia, while also supervising these initiatives. In collaborative interaction, the evolutionary game law of enterprises, academic institutions and governments reveals that the government formulates strong reward and punishment policies for industry-university-research cooperation projects, which helps establish an extensive and sustainable IUR network [1].

In the context of open innovation, an increasing number of stakeholders are becoming involved in the cooperation network. Apart from industry, academia, and government, science and technology intermediaries are playing a crucial role in IUR collaborations, particularly with the shift towards market-oriented approaches [20]. These intermediaries refer to the fourth independent or relatively independent agent of the enterprises, academic institutions and governments, including technology transfer institutions, financial investment institutions, professional intermediaries and digital platforms [21,22]. They provide support, catalysis or supply and demand matching services for cooperative innovation. Within IUR networks, science and technology intermediaries are pivotal in overcoming challenges such as knowledge disparities and conflicting objectives among different entities, thereby enhancing the efficacy of collaborative innovation and technology transfer [23].

Nevertheless, existing research on the IUR network has not thoroughly explored the interactions among industry, academia, government and technology intermediaries, overlooking their respective roles during the process of cooperation in a comprehensive view. From the perspective of government guidance and Keynesianism, some studies have concluded that the government, as a third party, can guide and stabilize the process of IUR cooperation [18]. Focusing on the interactions among multiple agents in the cooperation network, relevant literature mostly analyzes knowledge or technology transfer from research institutes to enterprises [4]. However, the respective roles of the four agents during their interactions—particularly the role of science and technology intermediaries as market-driven entities—require deeper examination to understand how they collectively facilitate value co-creation within complex innovation ecosystems.

### IUR value co-creation

This collective action is essential because technological innovation is becoming increasingly complex and systematic. Consequently, it is difficult for enterprises to independently undertake the continuous innovation supply of complex systems. Cooperating with multiple agents has become an effective way to improve the innovation capability of enterprises [24]. Regarding IUR cooperation, with the increasing number of participants and the formation of networks, the interaction among multiple agents has evolved from knowledge or technology transfer to value co-creation [9]. IUR value co-creation refers to the interactive behavior and process of multiple participants who collaboratively invest, share, and integrate resources to create value together and achieve scientific and technological innovation during cooperation [25,26]. In the IUR network, enterprises, academic institutions, government, and intermediaries collaborate to create value through cooperative patent incubation, entrusted R&D, joint R&D, and co-construction of research institutions.

IUR value co-creation is influenced by various factors, such as the willingness of key participants, the allocation of resources, and the management of the value co-creation process [27,28]. Furthermore, organizational goals and incentives influence the willingness to participate in value co-creation [10], which in turn affects the final performance of value co-creation. Among the intangible resources invested, participants’ qualifications, capabilities, and knowledge base are important factors determining the value co-creation’s effectiveness [29,30]. At the same time, in the process of collaboration, the IUR network, relying on the capabilities and resources of all parties, provides rich opportunities and effective support for value co-creation [31]. Besides, in the process of value co-creation, establishing and strengthening mutual trust among multiple agents, selecting appropriate intellectual property strategies, and constructing digital platforms are all stimuli for collaborators to integrate resources and create value [32].

Previous studies have discussed some influencers of value co-creation in IUR cooperation [33], but few studies have integrated value co-creation into the IUR cooperative network environment to reveal the interactive behavior of multiple agents and their respective roles [10]. Furthermore, value co-creation evolves in response to the roles and behaviors of agents within the IUR network [34]. However, the evolutionary patterns and dynamics of value co-creation have yet to be explored. Evolutionary games, which examine the dynamic equilibrium process of decision-making after continuous learning, imitation and adjustment, can effectively investigate the strategic dynamics and evolution of value co-creation [14]. Critically, while agent-based modeling simulates emergent complexity through predefined behavioral rules and econometric analysis identifies statistical associations from observational data, evolutionary game theory is uniquely suited to capture the core dynamics of IUR interactions—specifically modeling how boundedly rational agents continuously adapt their value co-creation strategies through payoff-driven learning, imitation of successful behaviors, and institution-level strategy evolution [35]. Therefore, this research adopts the evolutionary game methodology to deconstruct the value co-creation process from a multi-agent game perspective and analyze the influencing factors and measures for improving value co-creation within the IUR network.

### Analytical framework

**Fig 1:**
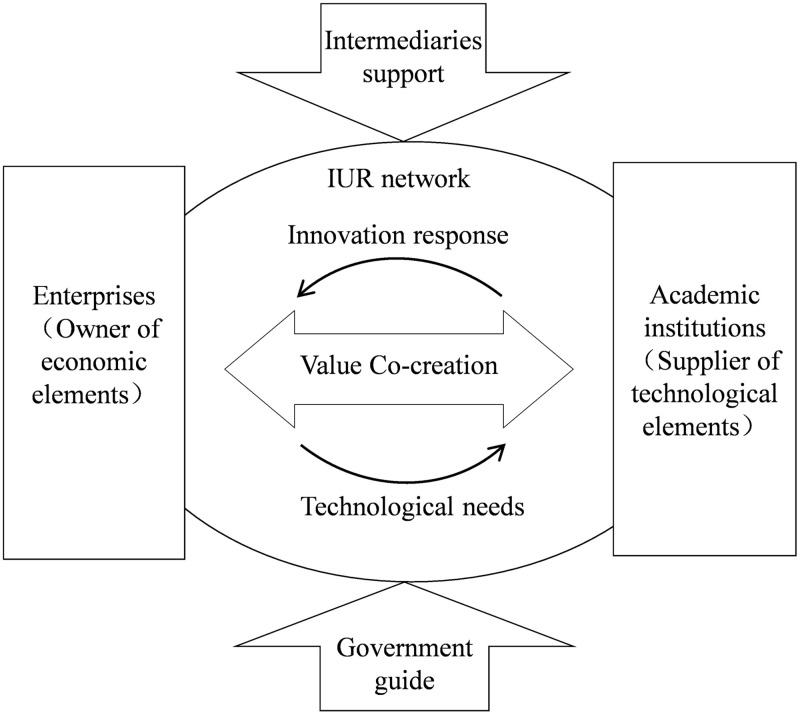
Analytical framework of value co-creation within the IUR network.

Value co-creation by multiple agents in the IUR network integrates heterogeneous advantageous resources and emphasizes risk and benefit sharing, maximizing the functional complementarity and operational coupling of the cooperation network to generate added value for the entire network. Fig 1 delineates the structured relationships among various agents to elucidate the value co-creation process. Enterprises, situated on the left side of the figure, function as the primary drivers and demanders of value co-creation. As owners of economic resources, they allocate capital, technology, information, and other assets. Their position in Fig 1 indicates their pivotal role in initiating the value co-creation process by articulating technological requirements, as indicated by the arrows extending from enterprises to academic institutions, which signify the transmission of these needs. Conversely, academic institutions, positioned on the right side of Fig 1, act as knowledge providers within the value co-creation framework. As suppliers of scientific and technological elements, they invest in talent and knowledge to advance research and development. The arrows directed from academic institutions to enterprises represent their innovative responses to the technological demands posed by enterprises. This bidirectional interaction between enterprises and academic institutions constitutes the core of the value co-creation process within the IUR network, as depicted by the double-headed arrows labeled “Value Co-creation,” “Innovation response,” and “Technological needs” at the center of the figure.

Beyond these principal agents, Fig 1 incorporates additional participants who indirectly facilitate value co-creation within the IUR network. Governments, located at the bottom of the figure, serve as policymakers guiding the value co-creation process. The arrows emanating from the government toward the IUR network symbolize the provision of financial incentives and the imposition of institutional constraints designed to direct the value co-creation activities. Science and technology intermediaries, positioned at the top of the figure, function as service providers supporting value co-creation. The arrows extending from these intermediaries to the IUR network denote the professional services they provide for innovation support and incubation.

The structural relationships depicted in Fig 1 directly support the study’s assumptions. Enterprises and academic institutions act as value co-creators within the IUR network. In the value co-creation process, enterprise contributions include R&D funding and technology, while academic institutions primarily provide knowledge and talent. As policy supporters, governments offer subsidies and enforce penalties. Additionally, science and technology intermediaries facilitate the process by enhancing the speed and quality of value co-creation within the network. Therefore, from a multi-agent perspective, this study uses Fig 1 as the analytical framework. It not only clearly shows the roles and relationships of different agents in the IUR network but also provides a logical foundation for establishing an evolutionary game model. In the evolutionary game model, the strategies and interactions of these agents, as depicted in Fig 1, can be further analyzed to understand how they evolve over time to achieve more efficient value co-creation in the IUR network.

## Evolutionary game model

### Assumptions and variables

Assumption 1: Enterprises and academic institutions are the value co-creators in the IUR network. This paper studies the two types of heterogeneous agents participating in the game of value co-creation centered on themselves or neighbors within the network. Under bounded rationality influenced by information asymmetry, agents update strategies according to the Fermi rule. At each evolutionary step, an agent randomly selects a neighbor, compares their payoffs, and adopts the neighbor’s strategy with a probability determined by Fermi Function. Through repeated interactions, the system converges toward evolutionary stable strategies. All agents possess binary strategy sets: {Positive co-creation; Negative co-creation}.

Assumption 2: In the process of value co-creation, the enterprise investment (I, I > 1) includes R&D funds, knowledge and technology. If the enterprise chooses the strategy of positive co-creation, its investment is I_1_. In negative co-creation, the enterprise investment is I_2_ (I_2 _< I_1_). The input of the academic institution (K, K > 1) mainly includes knowledge and talents. When choosing positive co-creation, the academic knowledge input is K_1_. when choosing negative co-creation, the input is K_2_ (K_2_ < K_1_). The investment or input of both parties equals to their costs respectively.

Assumption 3: As the policy supporter in value co-creation, the government provides subsidies (S) to each party if they both choose positive co-creation; If one party positively co-creates and the other party negatively co-creates, the negative co-creator is fined P and compensated to the positive co-creator, P∈(0, S); If both parties choose a negative co-creation strategy, there is no subsidy or punishment.

Assumption 4: As the service supporter in value co-creation, the science and technology intermediary promotes the transformation of IUR cooperative achievements into business (the transformation coefficient is A). In practice, enterprises hire science and technology service institutes and pay for service costs Cs.

Assumption 5: The payoff of cooperation in value co-creation is also determined by the enterprise’s knowledge absorption capacity coefficient α and the academic institution’s R&D capacity coefficient β. Referring to the Cobb-Douglas production function, considering the interaction between the enterprise and the academic institution, the benefit of IUR co-creation is AK^α^I^β^(0 < A < 1). The benefit distribution coefficients of enterprises and academic institutions are θ (0 < θ < 1) and 1-θ, respectively.

### Evolutionary game model

Based on the above assumptions, the payoff matrix of the game between enterprises and academic institutions in the process of value co-creation in the IUR network is shown in Table 1.

**Table 1:** Payoff matrix of value co-creation game in IUR network.

		University or research institute	
		Positive co-creation	Negative co-creation
Enterprise	Positive co-creation	θAK_1_^α^I_1_^β^－I_1_－C_s_ ＋ S	θAK_2_^α^I_1_^β^－I_1_－C_s_ ＋ P
		(1-θ)AK_1_^α^I_1_^β^－K_1_ ＋ S	(1-θ)AK_2_^α^I_1_^β^－K_2_－P
	Negative co-creation	θAk_1_^α^I_2_^β^－I_2_－C_s_－P	θAk_2_^α^I_2_^β^－I_2_－C_s_
		(1-θ)Ak_1_^α^I_2_^β^－K_1_ ＋ P	(1-θ)Ak_2_^α^I_2_^β^－k_2_

The value co-creators in the IUR network have bounded rationality. In the evolution process, they conduct multiple game interactions and strategy learning with neighbors, update strategy according to certain rules, and finally reach a stable equilibrium state. Assuming that in the initial state, the probability of enterprises adopting the positive co-creation strategy is x, and the probability of adopting the negative co-creation strategy is 1-x; the probability of adopting the positive co-creation strategy is y, and the probability of adopting the negative co-creation strategy is 1-y.

(1) The enterprise’s expected payoff and replication dynamic equation

The expected payoff of the enterprise choosing the positive co-creation strategy is


$$R{\mathrm{\backslash nolimits}}_{1}=y\cdot \left(\theta AK{\mathrm{\backslash nolimits}}_{1}^{\alpha }I{\mathrm{\backslash nolimits}}_{1}^{\beta }-I{\mathrm{\backslash nolimits}}_{1}-C{\mathrm{\backslash nolimits}}_{S}+S\right)+\left(1-y\right)\left(\theta AK{\mathrm{\backslash nolimits}}_{2}^{\alpha }I{\mathrm{\backslash nolimits}}_{1}^{\beta }-I{\mathrm{\backslash nolimits}}_{1}-C{\mathrm{\backslash nolimits}}_{S}+P\right)$$ (1)

The expected payoff of the enterprise choosing the negative co-creation strategy is


$$R{\mathrm{\backslash nolimits}}_{2}=y\cdot \left(\theta AK{\mathrm{\backslash nolimits}}_{1}^{\alpha }I{\mathrm{\backslash nolimits}}_{2}^{\beta }-I{\mathrm{\backslash nolimits}}_{2}-C{\mathrm{\backslash nolimits}}_{S}-P\right)+\left(1-y\right)\left(\theta AK{\mathrm{\backslash nolimits}}_{2}^{\alpha }I{\mathrm{\backslash nolimits}}_{2}^{\beta }-I{\mathrm{\backslash nolimits}}_{2}-C{\mathrm{\backslash nolimits}}_{S}\right)$$ (2)

It is concluded that the enterprise’s average expected payoff is


$$R=xR{\mathrm{\backslash nolimits}}_{1}+\left(1-x\right)R{\mathrm{\backslash nolimits}}_{2}$$ (3)

The enterprise’s replication dynamic equation is


f(x)=\raise0.7ex{dx\)/dxdt\nulldelimiterspace\lower0.7ex\({dt\)}}=x(R\nolimits1−R)=x(1−x\rightleft(R\nolimits1−R\nolimits2)=x(1−x){y[s+θA(K\nolimits1α−K\nolimits2α)(I\nolimits1β−I\nolimits2β)]+θAK\nolimits2α(I\nolimits1β−I\nolimits2β)+I\nolimits2−I\nolimits1+P} (4)

(2) The academic institution’s expected payoff and replication dynamic equation

The expected payoff of the academic choosing the positive co-creation strategy is


$$E{\mathrm{\backslash nolimits}}_{1}=x\cdot \left[\left(1-\theta \right)AK{\mathrm{\backslash nolimits}}_{1}^{\alpha }I{\mathrm{\backslash nolimits}}_{1}^{\beta }-K{\mathrm{\backslash nolimits}}_{1}+S\right]+\left(1-x\right)\left[\left(1-\theta \right)AK{\mathrm{\backslash nolimits}}_{1}^{\alpha }I{\mathrm{\backslash nolimits}}_{2}^{\beta }-K{\mathrm{\backslash nolimits}}_{1}+P\right]$$ (5)

The expected payoff of the academic choosing the negative co-creation strategy is


$$E{\mathrm{\backslash nolimits}}_{2}=x\cdot \left[\left(1-\theta \right)AK{\mathrm{\backslash nolimits}}_{2}^{\alpha }I{\mathrm{\backslash nolimits}}_{1}^{\beta }-K{\mathrm{\backslash nolimits}}_{2}-P\right]+\left(1-x\right)\left[\left(1-\theta \right)AK{\mathrm{\backslash nolimits}}_{2}^{\alpha }I{\mathrm{\backslash nolimits}}_{2}^{\beta }-K{\mathrm{\backslash nolimits}}_{2}\right]$$ (6)

It is concluded that the academic institution’s average expected payoff is


$$E=yE{\mathrm{\backslash nolimits}}_{1}+\left(1-y\right)E{\mathrm{\backslash nolimits}}_{2}$$ (7)

The academic institution’s replication dynamic equation is


g(y)=\raise0.7ex{dy\)/dydt\nulldelimiterspace\lower0.7ex\({dt\)}}=y(E\nolimits1−E)=y(1−y\rightleft(E\nolimits1−E\nolimits2)=y(1−y){x[s+(1−θ)A(K\nolimits1α−K\nolimits2α)(I\nolimits1β−I\nolimits2β)]+(1−θ)AI\nolimits2β(K\nolimits1α−K\nolimits2α)+K\nolimits2−K\nolimits1+P} (8)

### Analysis of evolutionarily stable strategies

From the previous analysis, the two-dimensional dynamic equation for IUR value co-creation is obtained as follows.


$$\left\{ \begin{array}{c} f(x)=\frac{dx}{dt}=x\left(1-x\right)\{y\left[s+\theta A\left(K{\mathrm{\backslash nolimits}}_{1}^{\alpha }-K{\mathrm{\backslash nolimits}}_{2}^{\alpha }\right)\left(I{\mathrm{\backslash nolimits}}_{1}^{\beta }-I{\mathrm{\backslash nolimits}}_{2}^{\beta }\right)\right]+\theta AK{\mathrm{\backslash nolimits}}_{2}^{\alpha }\left(I{\mathrm{\backslash nolimits}}_{1}^{\beta }-I{\mathrm{\backslash nolimits}}_{2}^{\beta }\right)+I{\mathrm{\backslash nolimits}}_{2}-I{\mathrm{\backslash nolimits}}_{1}+P\}\\ g(y)=\frac{dy}{dt}=y\left(1-y\right)\{x\left[s+\left(1-\theta \right)A\left(K{\mathrm{\backslash nolimits}}_{1}^{\alpha }-K{\mathrm{\backslash nolimits}}_{2}^{\alpha }\right)\left(I{\mathrm{\backslash nolimits}}_{1}^{\beta }-I{\mathrm{\backslash nolimits}}_{2}^{\beta }\right)\right]+\left(1-\theta \right)AI{\mathrm{\backslash nolimits}}_{2}^{\beta }\left(K{\mathrm{\backslash nolimits}}_{1}^{\alpha }-K{\mathrm{\backslash nolimits}}_{2}^{\alpha }\right)+K{\mathrm{\backslash nolimits}}_{2}-K{\mathrm{\backslash nolimits}}_{1}+P\} \end{array}\right.$$ (9)

When f (x) = 0, g (y) = 0, the system reaches five equilibrium points: (0,0), (0,1), (1,0), (1,1), and (x^*^, y^*^), where $x*=\frac{K{\mathrm{\backslash nolimits}}_{1}-K{\mathrm{\backslash nolimits}}_{2}-\left(1-\theta \right)AI{\mathrm{\backslash nolimits}}_{2}^{\beta }\left(K{\mathrm{\backslash nolimits}}_{1}^{\alpha }-K{\mathrm{\backslash nolimits}}_{2}^{\alpha }\right)-P}{S+\left(1-\theta \right)A\left(I{\mathrm{\backslash nolimits}}_{1}^{\beta }-I{\mathrm{\backslash nolimits}}_{2}^{\beta }\mathrm{\backslash rightleft}(K{\mathrm{\backslash nolimits}}_{1}^{\alpha }-K{\mathrm{\backslash nolimits}}_{2}^{\alpha }\right)}$, $y*=\frac{I{\mathrm{\backslash nolimits}}_{1}-I{\mathrm{\backslash nolimits}}_{2}-\theta AK{\mathrm{\backslash nolimits}}_{2}^{\alpha }\left(I{\mathrm{\backslash nolimits}}_{1}^{\beta }-I{\mathrm{\backslash nolimits}}_{2}^{\beta }\right)-P}{S+\theta A\left(I{\mathrm{\backslash nolimits}}_{1}^{\beta }-I{\mathrm{\backslash nolimits}}_{2}^{\beta }\mathrm{\backslash rightleft}(K{\mathrm{\backslash nolimits}}_{1}^{\alpha }-K{\mathrm{\backslash nolimits}}_{2}^{\alpha }\right)}$.

This paper only discusses the case when x^*^∈(0, 1) and y*∈(0, 1). Stability analysis is conducted using the following Jacobian matrix.


$$J=\left[\begin{array}{cc} {}*20c\frac{\partial f}{\partial x} & \frac{\partial f}{\partial y}\\ \frac{\partial g}{\partial x} & \frac{\partial g}{\partial y} \end{array}\right]=\left[\begin{array}{cc} {}*20ca{\mathrm{\backslash nolimits}}_{11} & a{\mathrm{\backslash nolimits}}_{12}\\ a{\mathrm{\backslash nolimits}}_{21} & a{\mathrm{\backslash nolimits}}_{22} \end{array}\right]$$ (10)

Where $a{\mathrm{\backslash nolimits}}_{11}=(1-2x\mathrm{\backslash rightleft}\{y\left[S+\theta A\left(K{\mathrm{\backslash nolimits}}_{1}^{\alpha }-K{\mathrm{\backslash nolimits}}_{2}^{\alpha }\right)\left(I{\mathrm{\backslash nolimits}}_{1}^{\beta }-I{\mathrm{\backslash nolimits}}_{2}^{\beta }\right)\right]+\theta AK{\mathrm{\backslash nolimits}}_{2}^{\alpha }\left(I{\mathrm{\backslash nolimits}}_{1}^{\beta }-I{\mathrm{\backslash nolimits}}_{2}^{\beta }\right)+I{\mathrm{\backslash nolimits}}_{2}-I{\mathrm{\backslash nolimits}}_{1}+P\}$, $a{\mathrm{\backslash nolimits}}_{12}=x\left(1-x\mathrm{\backslash rightleft}[S+\theta A\left(K{\mathrm{\backslash nolimits}}_{1}^{\alpha }-K{\mathrm{\backslash nolimits}}_{2}^{\alpha }\right)\left(I{\mathrm{\backslash nolimits}}_{1}^{\beta }-I{\mathrm{\backslash nolimits}}_{2}^{\beta }\right)\right]$, $a{\mathrm{\backslash nolimits}}_{21}=y\left(1-y\mathrm{\backslash rightleft}[S+\left(1-\theta \right)A\left(K{\mathrm{\backslash nolimits}}_{1}^{\alpha }-K{\mathrm{\backslash nolimits}}_{2}^{\alpha }\right)\left(I{\mathrm{\backslash nolimits}}_{1}^{\beta }-I{\mathrm{\backslash nolimits}}_{2}^{\beta }\right)\right]$,

$a{\mathrm{\backslash nolimits}}_{22}=(1-2y\mathrm{\backslash rightleft}\{x\left[S+\left(1-\theta \right)A\left(K{\mathrm{\backslash nolimits}}_{1}^{\alpha }-K{\mathrm{\backslash nolimits}}_{2}^{\alpha }\right)\left(I{\mathrm{\backslash nolimits}}_{1}^{\beta }-I{\mathrm{\backslash nolimits}}_{2}^{\beta }\right)\right]+\left(1-\theta \right)AI{\mathrm{\backslash nolimits}}_{2}^{\beta }\left(K{\mathrm{\backslash nolimits}}_{1}^{\alpha }-K{\mathrm{\backslash nolimits}}_{2}^{\alpha }\right)+K{\mathrm{\backslash nolimits}}_{2}-K{\mathrm{\backslash nolimits}}_{1}+P\}$.

Then the determinant Det(J) and the trace Tr(J) of the Jacobian matrix are calculated. An equilibrium point obtained from the dynamic equation is considered an Evolutionarily Stable Strategy (ESS) only if it satisfies both of the following conditions simultaneously: $Det\left(J\right)=a{\mathrm{\backslash nolimits}}_{11}a{\mathrm{\backslash nolimits}}_{22}-a{\mathrm{\backslash nolimits}}_{12}a{\mathrm{\backslash nolimits}}_{21}>0$, $Tr\left(J\right)=a{\mathrm{\backslash nolimits}}_{11}+a{\mathrm{\backslash nolimits}}_{22}<0$.

**Fig 2:**
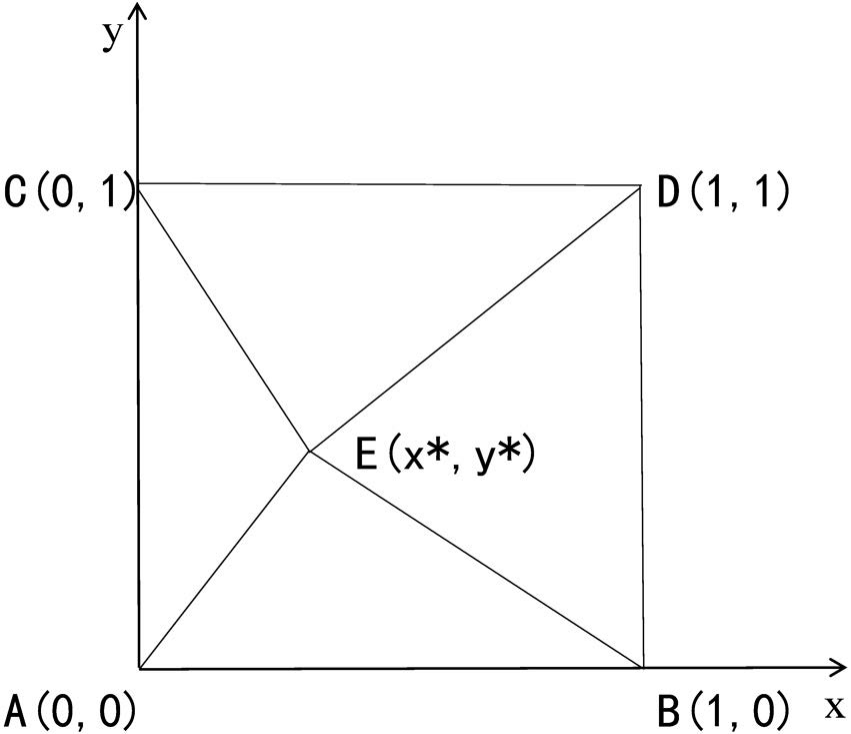
Evolutionary game phase diagram. A(0, 0): Det(J) > 0, Tr(J) < 0, stable point; B(1, 0): Det(J) > 0, Tr(J) > 0, unstable point; C(0, 1): Det(J) > 0, Tr(J) > 0, unstable point; D(1, 1): Det(J) > 0, Tr(J) < 0, stable point; E(x*, y*): Det(J) < 0, Tr(J) = 0, saddle point.

The stability results of the five equilibrium points and the dynamic evolution process of the players’ strategy choices are shown in Fig 2. The equilibrium points (0,0) and (1,1) represent the ESS in this case. The evolutionary phase diagram shown in Fig 2 illustrates that the stable strategies are either (positive co-creation, positive co-creation) or (negative co-creation, negative co-creation), depending on the probability that the agent initially chooses positive co-creation. If the system’s initial state is located in region BECD, the stable strategy of the evolutionary game is (positive co-creation, positive co-creation), indicating that a stable and positive co-creative relationship can be formed between the two parties. However, if the system’s initial state is located in region ABEC, the stable strategy of the evolutionary game is (negative co-creation, negative co-creation), meaning that the IUR co-creation relationship lacks long-term stability.

## IUR network construction and strategy updating

### IUR network construction

As a strategic emerging industry in China, the high-end equipment manufacturing industry produces high-tech equipment for related industries and national economy. It is characterized by high innovation, high added value, and strong industrial correlation. The high-end equipment manufacturing industry is considered the core of the manufacturing sector and the backbone of national economic development. It plays a crucial role in driving the transformation and upgrading of the entire manufacturing industry, serving as a key focal point for China’s “Made in China 2025” initiative. In this context, the high-end equipment manufacturing industry has become a key area for scientific and technological innovation as well as an important hub for collaboration between industry and academia. Enterprises are striving to enhance their technical capabilities and innovation through collaborative research efforts. Chinese high-end equipment manufacturing enterprises have established extensive cooperative relationships with universities and research institutes. According to data from China’s State Intellectual Property Office (SIPO), the number of patent applications of high-end equipment manufacturing industry has always been in the leading position among the patents jointly applied by industry and academia. Based on the patent application data of China’s high-end equipment manufacturing industry, this paper constructs its IUR cooperation network.

**Fig 3:**
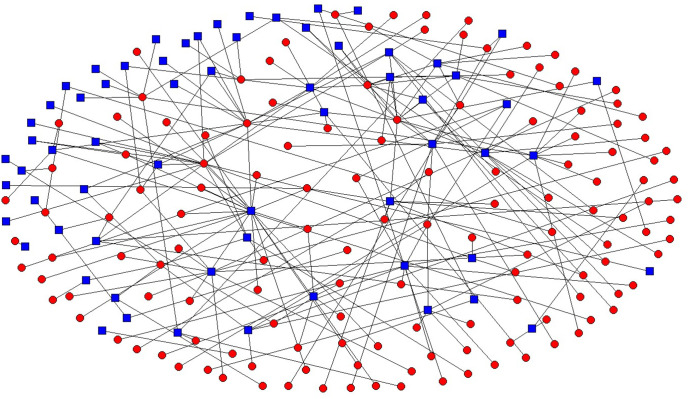
IUR network of China’s high-end equipment manufacturing industry.

For this study, patent searches were conducted using the PATSNAP platform with selection from SIPO’s patent database. The search formula was set to include current applicants (patent holders): (enterprise OR group OR factory) AND (university OR institute). In 2010, the classification of strategic emerging industries included the designation of the high-end equipment manufacturing sector within its scope. The high-end equipment manufacturing industry was selected in the strategic emerging industry classification, and the mechanical equipment was selected in the application field. The utility model was excluded from the patent type, resulting in 698 patent data being obtained. After conducting patent duplication and denoising based on the ranking of applicants and keywords of the high-end equipment manufacturing industry, a total of 540 patents were screened to construct an IUR network. Finally, UCINET software was used for visual processing to obtain the IUR network map of China’s high-end equipment manufacturing industry (see Fig 3). In this figure, each node represents a patent applicant, totaling 202 nodes: 138 red nodes representing enterprises and 64 blue nodes representing universities and research institutes. Each of the 221 edges represents a cooperative patent application relationship between the connected nodes. An edge weight of 1 indicates the presence of a connection between two nodes; if no connection exists, the edge weight is 0.

### Strategy updating

In the IUR network, the sum of the number of nodes that have a cooperation relationship with the node m is d_m_, which is the degree centrality. When constructing the network, this cooperation relationship is measured by a joint patent application. The set of all adjacent nodes with node m is N_m_ = {n_1_,n_2_,...,n_dm_}. All nodes in the set have a cooperation relationship and conduct a value co-creation game with node m.

The probability that node m chooses the positive co-creation strategy in time t is as follows.


$$P{\mathrm{\backslash nolimits}}_{m}\left(t\right)=\{\begin{array}{c} {}*20cx{\mathrm{\backslash nolimits}}_{m},\;m\;is\;an\;enterprise\\ y{\mathrm{\backslash nolimits}}_{m},\;m\;is\;an\;academic \end{array}\begin{array}{c} {}*20c \end{array}$$ (11)

The expected payoff of node m in time t is as follows.


$$U{\mathrm{\backslash nolimits}}_{m}\left(t\right)=\{\begin{array}{cc} {}*20c\sum {\mathrm{\backslash nolimits}}_{n\in N{\mathrm{\backslash nolimits}}_{m}}\left[x{\mathrm{\backslash nolimits}}_{m}R{\mathrm{\backslash nolimits}}_{1}^{m}+\left(1-x{\mathrm{\backslash nolimits}}_{m}\right)R{\mathrm{\backslash nolimits}}_{2}^{m}\right],\;m\;is\;an\;enterprise & \\ \sum {\mathrm{\backslash nolimits}}_{n\in N{\mathrm{\backslash nolimits}}_{m}}\left[y{\mathrm{\backslash nolimits}}_{m}E{\mathrm{\backslash nolimits}}_{1}^{m}+\left(1-y{\mathrm{\backslash nolimits}}_{m}\right)E{\mathrm{\backslash nolimits}}_{2}^{m}\right],\;m\;is\;an\;academic & \end{array}$$ (12)

In the IUR network, the node m plays a value co-creation game with all its adjacent nodes N_m_, and receives the payoff according to the matrix. R_1_^m^, R_2_^m^, E_1_^m^, and E_2_^m^ represent the expected payoffs of node m under different strategy combinations in the value co-creation game. Then, node m randomly selects the neighbor n in the network for comparison, learns the strategy of the neighbor node and imitates it with a certain probability W. Using Fermi function as the strategy update rule in the IUR network, the probability of the node m learning the node n’s strategy is


W\nolimitsn→m=11+exp[(U\nolimitsn(t)d\nolimitsn−U\nolimitsm(t)d\nolimitsm)/(U\nolimitsn(t)d\nolimitsn−U\nolimitsm(t)d\nolimitsm)k\nulldelimiterspacek] (13)

where $\frac{U{\mathrm{\backslash nolimits}}_{m}\left(t\right)}{d{\mathrm{\backslash nolimits}}_{m}}$ or $\frac{U{\mathrm{\backslash nolimits}}_{n}\left(t\right)}{d{\mathrm{\backslash nolimits}}_{n}}$ is the average expected revenue of node m or n in time t. k (k > 0) means the noise, which represents the irrational degree of the agent. When k is approaching 0, the noise is 0 and the agent is completely rational. In this case, if the average expected revenue of node n is higher than that of node m, node m will almost surely imitate the strategy of node n. When k is approaching ∞ , the information is submerged by noise and the agent is completely irrational. A higher k value means that the agent is more likely to make random strategy changes regardless of the difference in expected revenues. Referring to the reality and extant research, 0.5 is chosen as the k value in the game simulation.

According to the strategy updating rule, the system reaches a mixed equilibrium when the expected payoffs of positive and negative co-creation strategy for a subset of nodes become equal, resulting in the stable coexistence of both strategies.

The probability that node m chooses the positive co-creation strategy in time t + 1 is


$$P{\mathrm{\backslash nolimits}}_{m}\left(t+1\right)=\left(1-W{\mathrm{\backslash nolimits}}_{n\to m}\right)P{\mathrm{\backslash nolimits}}_{m}\left(t\right)+W{\mathrm{\backslash nolimits}}_{n\to m}P{\mathrm{\backslash nolimits}}_{n}\left(t\right)$$ (14)

In the IUR network G = (V, E), V represents the set of all nodes in the network, and E represents the set of all edges in the network. In the network, the average probability of all nodes choosing positive co-creation strategy can reflect the evolutionary state of the value co-creation game. Therefore, as an indicator to measure the value co-creation effect, the average probability of positive co-creation in the network is


$$\overset{―}{p}=\underset{t\to \infty }{\lim}\frac{\sum {\mathrm{\backslash nolimits}}_{m\in V}p{\mathrm{\backslash nolimits}}_{m}\left(t\right)}{n}$$ (15)

## Numerical simulation analysis

Using MATLAB software, this paper simulates the network evolutionary game between enterprises and academic institutions within China’s high-end equipment manufacturing industry from value co-creation perspective. The specific algorithm steps and benchmark parameter values are set as follows:

Firstly, within the constructed IUR network of China’s high-end equipment manufacturing industry, positive co-creation and negative co-creation strategies are randomly assigned to each node. The number of game cycles is defined as T = 100, with an initial average probability of each node choosing a positive co-creation strategy set at 50% when t = 0.

Secondly, based on previous research [1,14,35] and the actual distribution of benefits during the game process within China’s high-end equipment manufacturing industry IUR network, benchmark parameters for the model are determined: I_1 _= 10, I_2_ = 5, and α = 1; K_1_ = 8, K_2_ = 4, and β = 1; S = 3, P = 1; Cs = 0.5, A = 0.2; θ = 0.5. All parameters used in the simulations (I₁, I₂, α, K₁, K₂, S, P, C_s_, A, θ) are defined in the section of Assumptions and variables.

Thirdly, at time t, node m engages in a game with its neighbors and compares the payoffs within the network. Subsequently, the strategy is updated according to the Fermi update rule, resulting in p_m_ (t + 1), representing the node’s positive co-creation probability at time t + 1. Then all nodes’ average positive co-creation probability is calculated after a new round of strategy updates.

Furthermore, to strengthen the robustness of the model, this research undertakes a series of additional simulation plots that illustrate the convergence process starting from diverse initial conditions. In these simulations, we specifically set up three distinct initial states by varying the initial values of two crucial parameters, the enterprise’s knowledge absorption capacity coefficient α and the academic institution’s R&D capacity coefficient β. These parameters play significant roles in shaping the evolutionary dynamics of the system under study. Three initial conditions were set as α = 0.5, β = 1; α = 1, β = 0.5 and α = 0.5, β = 0.5. By simulating the system under these different initial conditions, we can observe how the average positive co-creation probability evolves over time. Overall, the evolutionary trends of the average positive co-creation probability under these conditions exhibit consistency. The visualization of the simulation results is attached in supporting information. This consistency provides evidence for the robustness of our model, indicating that the model’s outcomes are not sensitive to the initial values of these key parameters and can reach stable states regardless of different initial conditions.

### Numerical simulation analysis of the enterprise

#### The enterprise investment.

**Fig 4:**
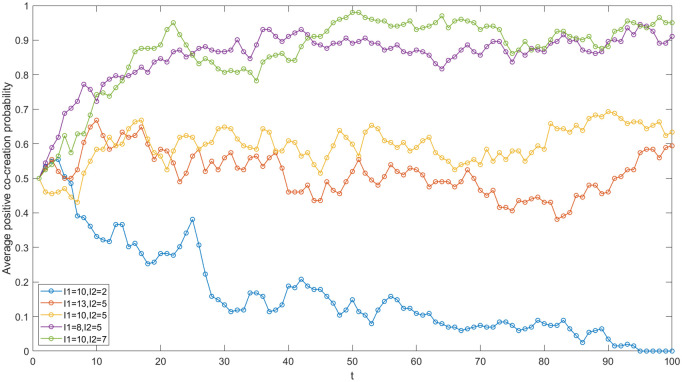
Evolutionary trajectories under varied enterprise investments.

The enterprises in the IUR network of China’s high-end equipment manufacturing industry include State Grid Co., Ltd., China National Offshore Oil Corporation, Shenyang Blower Group Co., Ltd and so on. In order to study the impact of enterprise investment on the value co-creation game in the network, five sets of values of I_1_ and I_2_ are set (see Fig 4) and other parameters are kept constant. Then, the simulation is run using MATLAB software, and the evolution trends in five cases are compared.

First and foremost, the smaller the difference between I_1_ and I_2_, the greater the average positive co-creation probability across all nodes in the network. According to the simulation results presented in Fig 4, when the differences between I_1_ and I_2_ are 3, 5, and 8, respectively, the average positive co-creation probability of the network gradually decreases. This indicates that the difference between I_1_ and I_2_ is negatively correlated with the average positive co-creation probability of the network. The primary reason for this trend is the consideration of cost savings and hitchhiking. Larger differences compel enterprises to favor a negative co-creation strategy due to the higher costs and risk. associated with positive co-creation investments compared to negative ones, which is consistent with the risk aversion theory [5]. Furthermore, the strategy learning among nodes contributes to a decline in the average positive co-creation probability of academic institutions and, by extension, the entire network. In the context of China’s high-end equipment manufacturing industry, the investment required for innovation is substantial, and the payback period is lengthy. Additionally, uncertainties such as the international situation contribute to the high risk associated with innovation projects in this sector. Consequently, during the process of IUR value co-creation, particularly in fundamental research projects, enterprises often exhibit hesitance in their investment decisions if achieving the desired payoff necessitates significant financial outlay.

Secondly, the increase in I_2_ contributes to the evolution of game strategy in the IUR network towards a positive co-creation direction. In Fig 4, when I_1_ = 10 and I_2_ = 2, 5, 7, the average probability of positive co-creation within the network increases. It is evident that there is a positive correlation between I_2_ and the average probability of positive co-creation in the network. The primary reason for this is that as I_2_ increases, the costs saved by enterprises opting for negative co-creation decrease. Additionally, compared to negative co-creation, a positive co-creation strategy can yield greater benefits in terms of achievement transformation. Consequently, enterprises are more inclined to adopt a positive co-creation strategy, which in turn encourages academic institutions to engage in positive co-creation, thereby enhancing the average probability of positive co-creation within the network. In the IUR network of China’s high-end equipment manufacturing industry, when enterprises and academic institutions sign cooperation agreements, they should establish a reasonable baseline for R&D investment and provide adequate financial support for cooperative innovation, facilitating value creation in a more favorable direction.

Thirdly, there is a negative correlation between I_1_ and the average probability of positive co-creation within the network. When I_2_ is set at 5 and I_1_ takes on the values of 8, 10, and 13, as illustrated in Fig 4, the average probability of positive co-creation within the network demonstrates a downward trend. It is evident that when I_2_ remains constant, a decrease in I_1_ leads to an increase in the average probability of positive co-creation within the network. In the context of China’s high-end equipment manufacturing industry’s Innovation and IUR project, the diminishing marginal revenue effect and the absence of a cooperation supervision mechanism imply that a continuous increase in R&D investment does not necessarily yield a corresponding improvement in cooperation payoffs. Instead, establishing a cooperative relationship based on mutual trust and a win-win guarantee mechanism is crucial. It is significant for high-end equipment manufacturing enterprises and academic institutions to effectively utilize R&D investments within the IUR network. This approach can reduce the enterprise investment required to achieve excess returns, thereby fostering positive value co-creation.

#### The enterprise’s knowledge absorption capacity coefficient.

**Fig 5:**
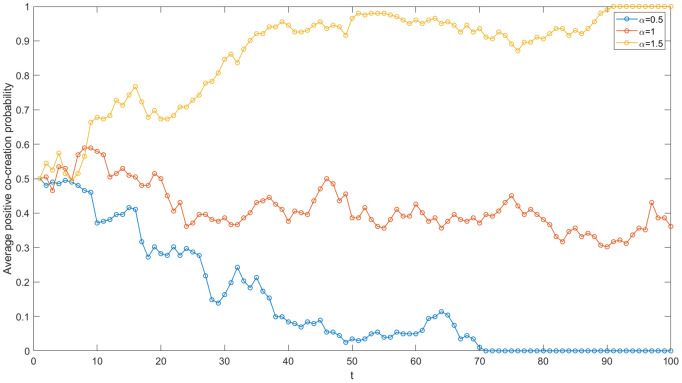
Evolutionary trajectories under varied enterprise’s knowledge absorption capacities.

The key to value co-creation within the IUR network lies in the process of generating new knowledge and delivering value through knowledge sharing between enterprises and academic institutions. During the knowledge creation and transfer process, enterprises assimilate the knowledge provided by academic institutions. Consequently, the knowledge absorption capacity of enterprises is critically important. As illustrated in Fig 5, when α = 0.5, the average positive co-creation probability of nodes in the network approaches 0. Conversely, when α = 1.5, it reaches 1. This indicates that the coefficient of enterprise knowledge absorption capacity is positively correlated with the average positive co-creation probability within the IUR network. From the knowledge-based view, the stronger an enterprise’s knowledge absorption capacity, the more likely it is that both enterprises and academic institutions will opt for a positive co-creation strategy. Consequently, the overall probability of positive co-creation within the entire network can be enhanced. For instance, China’s high-end equipment manufacturing enterprises, such as Shenyang Blower Group Co., Ltd., which supplies core equipment for the petroleum, chemical and other sectors, have relied on the introduction, digestion, and absorption of advanced foreign technologies to achieve product re-innovation during their early development stages. Additionally, these enterprises have absorbed and transferred new knowledge through multi-party cooperative innovation with academic institutions, ultimately leading to continuous breakthroughs in key core technologies and industry-wide common technologies. Knowledge absorption capacity has significantly fostered cooperative innovation among high-end equipment manufacturing enterprises and improved the value co-creation performance of IUR cooperation in China’s high-end equipment manufacturing industry. To enhance this capacity, policies could include offering tax incentives for R & D investment, providing subsidies for staff training on new knowledge, and promoting deeper enterprise-academia cooperation through joint projects and knowledge-sharing platforms.

#### The enterprise’s benefit distribution coefficient.

**Fig 6:**
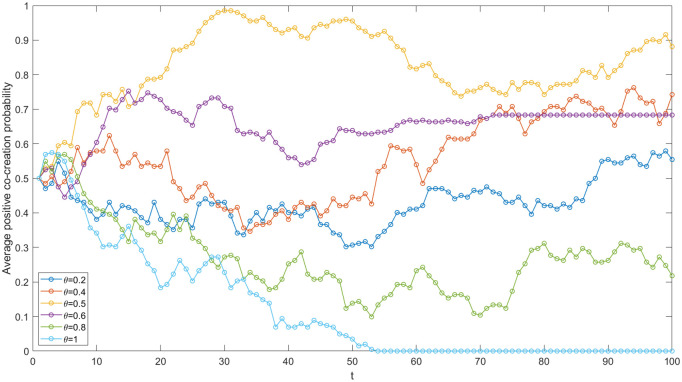
Evolutionary trajectories under varied enterprise’s benefit distribution coefficients.

A reasonable benefit distribution system is essential for the stable development of value co-creation in the IUR network. To investigate the influence of an enterprise’s benefit distribution coefficient on value co-creation within this network, this paper establishes six typical θ values while keeping other benchmark parameters constant in the game simulation. The results are illustrated in Fig 6. An inverted U-shaped relationship exists between the enterprise’s benefit distribution coefficient and the average positive co-creation probability. When θ is set at 0.5, the average positive co-creation probability reaches 0.9, approaching its maximum value. This suggests that a fair benefit distribution is conducive to promoting value co-creation within the IUR network, which is also supported by the equity rationale. Conversely, when the benefit distribution between the two parties is significantly uneven (θ = 0.8 or 1), the continuous cooperative game leads to a decrease in the positive co-creation probability for the party receiving fewer benefits during the strategy update process, thereby reducing the overall average positive co-creation probability of the network. During the IUR cooperation in China’s high-end equipment manufacturing industry, most enterprises pay a certain fee to the academic institutions, and the main benefits of the innovations are owned by the enterprises. However, the disparity of the benefit distribution cannot lead to ideal cooperation effect and technology transformation. With the mode of co-construction of research institutions, innovative achievements can be shared by both parties. Enterprises enjoy patent ownership and industrialization benefits. Universities can utilize the achievements to publish papers and apply for funds and professional titles. The fair benefit distribution mechanism facilitates value co-creation in the IUR network.

In the IUR network, enterprises are expected to take on a leading role in value co-creation activities. Enterprises can effectively create economic and social value with their partners by exercising reasonable control and supervision over enterprise investment, enhancing knowledge absorption capacity, and establishing an equitable benefit distribution mechanism. For instance, within China’s high-end equipment manufacturing industry IUR network, China Shipbuilding Heavy Industry Group (CSHIG) established independent research funds to formalize enterprise investment in IUR cooperation and developed internal research teams to enhance knowledge absorption capacity. This approach led to positive value co-creation. Additionally, CSHIG collaborated with the University of Science and Technology of China to establish the Quantum Joint Laboratory where research achievements are shared between both parties -the university gains non-economic benefits such as paper publication opportunities, fund applications, and title evaluations while CSHIG obtains patent ownership and achievement transformation benefits. This benefit distribution mechanism reduces transaction costs associated with collaborative innovation improves interest alignment between both parties, and ultimately promotes their value co-creation.

### Numerical simulation analysis of the academic

#### The academic knowledge input.

**Fig 7:**
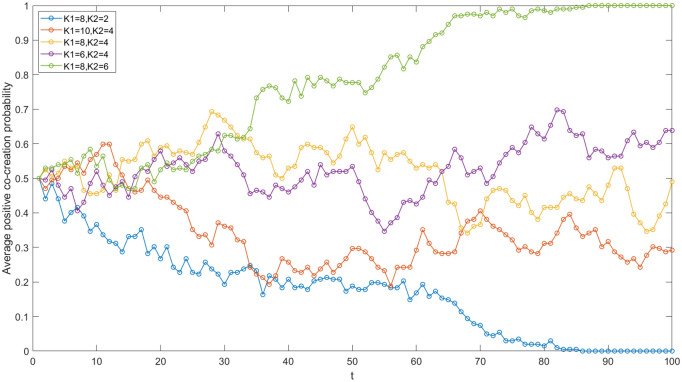
Evolutionary trajectories under varied academic knowledge inputs.

In the IUR network of China’s high-end equipment manufacturing industry, the academic institutions primarily include Tsinghua University, China University of Petroleum, South China University of Technology, the Chinese Academy of Sciences, and the China Academy of Railway Science. During the process of IUR value co-creation, the academic knowledge input also constitutes a significant cost. To investigate the influence of academic knowledge input on value co-creation, this paper establishes five sets of values for K_1_ and K_2_ (see Fig 7) while keeping other parameter values constant, and runs simulations using the software to obtain results.

Firstly, the smaller the difference between K_1_ and K_2_, the more favorable it is for increasing the average probability of positive co-creation within the network. In Fig 7, as the difference between K_1_ and K_2_ increases from 2 to 4–6, the average positive co-creation probability of the network decreases.

It can be inferred that a smaller disparity in knowledge input between negative co-creation and positive co-creation facilitates the adoption of positive co-creation strategies. This is because the cost savings academic institutions achieve by adopting a negative co-creation strategy are limited. In contrast, choosing a positive co-creation strategy can offer additional benefits to these institutions. As the gap between K_1_ and K_2_ narrows, the likelihood of academic institutions opting for positive co-creation increases, which subsequently raises the probability of enterprises also choosing positive co-creation. According to the knowledge transfer and innovation theory [17], the narrow gap between K_1_ and K_2_ can facilitate efficient knowledge flow and smooth transfer, leading to the increase in positive co-creation. Consequently, the variance in knowledge input between negative and positive co-creation by academic institutions demonstrates a negative correlation with the average probability of positive co-creation within the network. In China’s high-end equipment manufacturing industry, particularly in fundamental research projects, academic institutions are required to make sustained investments in knowledge. Achieving ideal outcomes through positive co-creation necessitates substantial input—especially when an institution’s knowledge base is inadequate—it may lead to principal-agent issues, such as opportunism and subpar performance. This is why a bigger gap between K_1_ and K_2_ can lead to negative co-creation in the industry.

Secondly, a higher level of academic knowledge input (K_2_) in negative co-creation is associated with a greater average probability of positive co-creation within IUR networks. As illustrated in Fig 7, maintaining K_1_ at 8 while incrementally increasing K_2_ from 2 to 4 and then to 6 leads to successive increases in the average probability of positive co-creations. It is evident that K_2_ has a positive correlation with this average probability within IUR networks. When the knowledge input from academic institutions is high in negative co-creation, the cost savings associated with this choice will be limited. Consequently, academic institutions are more likely to opt for positive co-creation, as this strategy can yield greater benefits. By adopting the strategies of neighboring entities within the network, enterprises will also enhance the likelihood of selecting a positive co-creation strategy, thereby improving the overall average probability of positive co-creation across the entire network. Therefore, it is essential to establish clear quantitative requirements for knowledge input and outcomes in the IUR cooperation agreements within the high-end equipment manufacturing industry. This is necessary to raise the minimum threshold for knowledge input from academic institutions, prevent free-riding behavior, and foster value co-creation within the IUR network.

Thirdly, K_1_ is found to have a negative correlation with the average probability of positive co-creation within the network. As illustrated in Fig 7, when K_2_ remains constant at 4 and K_1_ increases from 6 to 8 and then to 10, a general downward trend is observed in the average probability of positive co-creation within the network. It is evident that as K_1_ increases while K_2_ remains constant, the disparity between K_1_ and K_2_ widens. Consequently, although positive co-creation yields greater benefits with higher values of K_1_, negative co-creation tends to result in more significant cost savings. Therefore, academic institutions are more likely to adopt a negative co-creation strategy, which will lead to a decrease in the average probability of positive co-creation within the network. To address this issue within the IUR cooperation network of China’s high-end equipment manufacturing industry, it is essential for academic institutions to implement effective incentive mechanisms and constraints for talent. Additionally, establishing reasonable reward policies based on achievements and providing opportunities for mutual learning within the network can enhance innovation enthusiasm and efficiency among scientific research personnel, thereby ensuring the effective utilization of knowledge inputs.

#### The academic institution’s R&D capacity coefficient.

**Fig 8:**
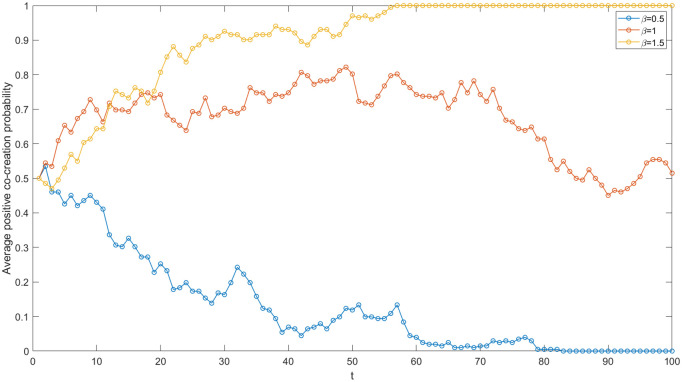
Evolutionary trajectories under varied academic institution’s R&D capacity coefficients.

In the context of IUR cooperation, academic institutions leverage the funds, information, technology, and other resources provided by enterprises to generate new knowledge based on their own R&D capabilities. Simultaneously, they share and transfer knowledge with these enterprises, ultimately achieving value co-creation. Consequently, the R&D capacity of academic institutions plays a pivotal role in the value co-creation process. In order to analyze the mechanisms of influence, this paper establishes three cases regarding the R&D capacity coefficient of academic institutions and simulates the network evolutionary game. The results are presented in Fig 8. When β = 0.5, the average positive co-creation probability of the network gradually approaches 0. When β = 1, the average positive co-creation probability fluctuates between 0.4 and 0.8. When β = 1.5, the average positive co-creation probability gradually increases to 1. The increase in the academic institution’s R&D capacity coefficient significantly enhances the average probability of positive co-creation within the network. The greater the R&D capacity of an academic institution, the more benefits agents anticipate from adopting a positive co-creation strategy. Consequently, the average probability of positive co-creation within the network is enhanced. The R&D capacity of the academic institution is essential for value co-creation within the IUR network in the high-end equipment manufacturing industry.

The network is primarily dominated by Tsinghua University, China University of Petroleum, the Institute of Oceanography, and the Chinese Academy of Sciences, along with other academic institutions that possess robust R&D capabilities. These institutions drive industry enterprises to create value and foster breakthroughs in essential common technologies. When selecting IUR partners, enterprises tend to prioritize academic institutions that have strong R&D capacities and a solid knowledge base in this field. Academic institutions serve as the custodians of scientific and technological resources and are the primary providers of knowledge within the IUR network. Consequently, the knowledge input and R&D capabilities of these institutions directly influence the efficiency of value co-creation.

### Numerical simulation analysis of the government

#### The government’s subsidy.

**Fig 9:**
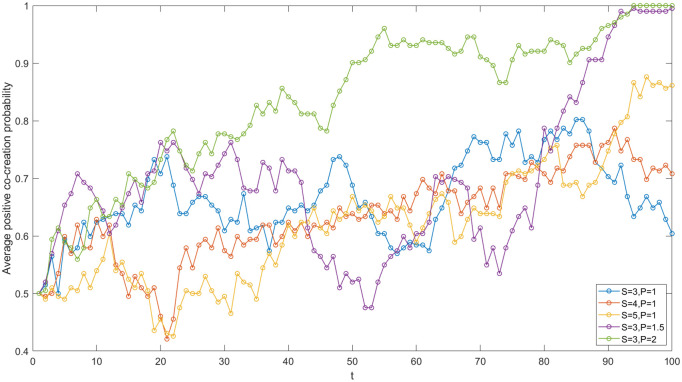
Evolutionary trajectories under varied government’s subsidies and penalties.

The government ‘s policy guidance on IUR cooperation has significantly enhanced the value co-creation between enterprises and academic institutions. Firstly, the government provides subsidies or rewards to enterprises and academic institutions that engage in value co-creation. This not only alleviates the financial burden associated with R&D investments but also mitigates the R&D risks faced by enterprises to some extent. Furthermore, these subsidies send positive signals to the external environment, which can attract financing opportunities and favorable conditions for collaborative projects, particularly for high-risk basic research initiatives with long development cycles. Consequently, an increase in government subsidies is likely to facilitate positive value co-creation. In order to study the effect of government subsidies on the IUR value co-creation, this paper keeps other parameter values constant, and sets three groups of government subsidies (S = 3, 4, 5) for simulation. The results are shown in Fig 9. When S = 3 and P=1, the average positive co-creation probability within the network gradually evolves to 0.60; When S = 4 and P=1, the average positive co-creation probability gradually evolves to 0.70; When S = 5 and P=1, the average positive co-creation probability gradually evolves to 0.86. It can be seen that the government’s subsidy is positively correlated with the average positive co-creation probability within the IUR network. Because of the increase of subsidies, the cooperators’ R&D cost required for positive co-creation is reduced. They will be inclined to obtain higher cooperative benefits through positive co-creation. With each other ‘s strategic learning, the average positive co-creation probability of all nodes within the network increases accordingly.

#### The government’s penalty.

Moreover, the government’s supervision and restraint policies play a significant role in the IUR value co-creation. Among these policies, the fine system is the most typical form of supervision and restraint. Therefore, the negative co-creator is assumed to be fined an amount P and this fine is then compensated to the positive co-creator. By controlling other parameters and establishing three groups with varying P values for simulation, the impact of different penalties on value co-creation in the IUR network is analyzed. The results presented in Fig 9 indicate that the average probability of positive co-creation in the network significantly improves as P increases (S = 3, P = 1, 1.5, 2). In comparison to the effect of subsidies (S), the increase in penalties (P) has a more pronounced positive effect on value co-creation within the network. The increase in punitive measures raises the costs associated with negative co-creation, discourages hitchhiking in value co-creation, and simultaneously compensates for the losses incurred by the positive co-creators. Consequently, agents in the network are more likely to adopt a positive co-creation strategy. Furthermore, in practice, government penalties for negative co-creators often involve public criticism and the revocation of subsidy eligibility. According to the regulatory and signaling theory [14], these actions send negative signals to external stakeholders, adversely affecting the organization’s image and long-term benefits, thereby further elevating the costs associated with negative co-creation strategies. As a result, due to the direct and indirect impacts of government restraint policies, cooperative agents within the network exhibit a heightened sensitivity to punishment, with the intensity of punishment being significantly positively correlated with the average probability of positive co-creation within the network.

The government serves as a policy supporter within the IUR network, promoting value co-creation through subsidies or penalties. The risks associated with IUR cooperation projects in China’s high-end equipment manufacturing industry are significant, necessitating substantial investment. Government subsidy policies can alleviate financial pressures and foster value co-creation. Particularly in the early stages of IUR cooperation, trust among various stakeholders is often lacking; thus, government involvement can guide enterprises and academic institutions toward positive interactions. Furthermore, the government’s establishment of policies and regulations, especially binding ones, can mitigate opportunism in IUR cooperation and lower transaction costs related to negotiation, implementation, and supervision.

### Numerical simulation analysis of the intermediary

#### The intermediary’s transformation coefficient.

**Fig 10:**
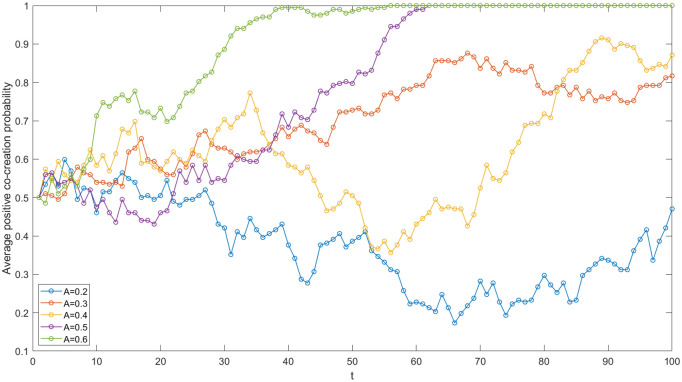
Evolutionary trajectories under varied intermediary’s transformation coefficients.

Science and technology intermediaries provide essential support services, information dissemination, intellectual property management, trading, and technical consulting for enterprises and academic institutions. These services facilitate effective connections between the supply and demand sides of scientific and technological achievements, thereby enhancing the IUR achievement transformation and fostering value co-creation as the value matching theory [22] confirms. In order to study the specific impact of science and technology intermediary’s service quality on value co-creation in the IUR network, this study simulates the network evolutionary game process using MATLAB software. The intermediary’s transformation coefficient (A), which reflects the service quality, was tested, and finally five groups of A values (see Fig 10) were selected to obtain the simulation results. In Fig 10, with the increase of A, the average positive co-creation probability of the network generally shows an upward trend. When A is 0.5 or 0.6, the average positive co-creation probability of the network gradually evolves to 1. This confirms a positive correlation between the transformation coefficient and the network’s average positive co-creation probability.

The higher transformation coefficient, which means that these intermediaries are more effective in their services, brings three main advantages leading to the increased positive co-creation. Firstly, it reduces the barriers to commercialization. Intermediaries providing efficient services can quickly match the technologies developed by academic institutions with the market demands of enterprises, cutting down the time and cost of commercialization. Secondly, a higher transformation coefficient implies that intermediaries can better reduce information asymmetry. They can provide accurate and timely information to both parties, enabling them to make more informed decisions and cooperate more effectively. Finally, intermediaries can promote long-term and stable co-creation relationship by establishing a good communication and cooperation mechanism between enterprises and academic institutions. They can help resolve potential conflicts and disputes by the cooperation agreement, creating a favorable environment for continuous positive value co-creation in the IUR network.

In the IUR network of China’s high-end equipment manufacturing industry, science and technology intermediaries encompass technology transfer institutions, financial investment entities, professional intermediaries, and digital platforms. While government guidance is crucial for the initial development of IUR cooperation, it is insufficient to ensure sustainable coordination, and thus a synergistic combination of market mechanisms and government guidance is essential to continuously promote value co-creation among diverse agents within the IUR network. The establishment of an effective science and technology intermediary system facilitates the value co-creation of the IUR network within China’s high-end equipment manufacturing industry. To optimize intermediary services in the IUR network, policymakers should: (1) Establish dedicated funding for science and technology intermediaries, implementing performance-anchored models tied to commercialization targets while supporting digital infrastructure modernization and cross-disciplinary broker training; (2) Deploy tiered fiscal incentives—including tax reductions—for market-driven intermediaries achieving benchmarked technology transfer volumes; (3) Create a merit-based accreditation ecosystem with national service standards and mandatory data-sharing consortia that integrate patent, manufacturing, and financial databases to eliminate information asymmetries and accelerate technology-to-market timelines.

## Conclusion

### Discussion

Based on the analysis framework of value co-creation in the IUR network, this paper constructs an evolutionary game model of value co-creation from the perspective of multiple agents. According to the IUR network of China’s high-end equipment manufacturing industry and the value co-creation practice, the game simulation and result analysis are conducted using MATLAB software. The key findings are as follows:

(1) Enterprises, as primary drivers and demanders of value co-creation, exhibit behaviors consistent with risk aversion theory. The simulation results reveal a negative correlation between the investment disparity and the average probability of positive co-creation, indicating that larger gaps increase perceived partnership risks. This finding aligns with previous research in strategic behavior, where participants tend to avoid the perceived uncertainties in partnerships [36]. When the investment disparity between positive and negative co-creation strategies is substantial, enterprises become more cautious about potential losses, thereby reducing the likelihood of engaging in positive co-creation. Furthermore, our study corroborates insights from the knowledge-based view and equity rationale. Enhancing enterprises’ knowledge absorption capacity and implementing an equitable benefit distribution mechanism significantly facilitate positive value co-creation. Enterprises with stronger knowledge absorption capabilities can more effectively leverage knowledge resources from partners, while an equitable benefit distribution system ensures all parties feel fairly treated, motivating more active contributions. Compared to prior studies that primarily focused on overall investment [3,8,9], our research highlights the importance of investment balance and knowledge absorption in the co-creation process.
(2) Based on knowledge transfer and innovation theory, academic institutions act as knowledge suppliers in the value co-creation process. The simulation results reveal a negative correlation between the disparity in academic’s knowledge inputs and the average probability of positive co-creation. This can be explained by the theory’s emphasis on efficient knowledge flow. A significant disparity between positive and negative co-creation knowledge inputs from the supply side may disrupt the smooth transfer and efficient flow of knowledge, as highlighted by previous studies [17,18] on knowledge diffusion in innovation networks. When academic institutions must contribute substantially more knowledge to achieve positive value co-creation, it may result in knowledge bottlenecks or misunderstandings, thereby hindering the co-creation process. Furthermore, the R&D capacity of academic institutions is positively correlated with value co-creation within the IUR network. This finding aligns with the general understanding that stronger R&D capabilities enable academic institutions to generate higher-quality knowledge, which is essential for value co-creation. However, unlike previous research [19,34] that primarily measured R&D capacity by the number of research projects or publications, our study considers the actual impact of R&D capacity on the co-creation process.
(3) From the perspective of regulatory and signaling theory, the government functions as a policy supporter. Simulation results indicate that increases in government subsidies and penalties positively influence value co-creation within the IUR network, suggesting that government policies can effectively send signals and regulate behavior. Government subsidies provide financial incentives for enterprises and academic institutions to engage in value co-creation, while penalties deter non-cooperative behaviors. The signaling effect of the punishment mechanism is particularly significant. Co-creators are highly sensitive to penalties, as these signal the government’s commitment to maintaining a healthy co-creation environment. This finding aligns with previous research [1,10] on the role of government policies in innovation ecosystems. However, our study further investigates the quantitative relationship between government policies and positive IUR value co-creation.
(4) According to market mechanism and value matching theory, science and technology intermediaries, as service supporters in IUR co-creation, help align supply and demand through market-oriented mechanisms, reduce information asymmetry, and foster sustainable development within the IUR network. This research reveals that the efficiency of science and technology intermediaries in matching the needs of enterprises and academic institutions is positively correlated with the average probability of positive co-creation. Previous research [3] has also explored the role of intermediaries in innovation networks, showing that their role is to bridge the gap between different agents, facilitating the flow of information, resources, and knowledge, which is supported by our study. Besides, we further analyze and define the intermediaries’ transformation coefficient that affects their efficiency. This coefficient highlights that improvements in intermediary service quality are effective means to enhance value co-creation within the IUR network.

These findings collectively validate the IUR network as a complex adaptive system in which agent interactions generate emergent co-creation patterns, as depicted by IUR co-creation analytical framework. In the IUR network system, enterprises initiate the value co-creation process by articulating technological requirements and providing economic resources. Their knowledge absorption capacity serve as a facilitating factor in this process. Meanwhile, academic institutions respond to enterprises’ needs by investing in knowledge and technological elements. Their strong R&D capabilities significantly enhance the value co-creation process. The government directs value co-creation activities by offering financial incentives and imposing institutional constraints, while efficient intermediaries facilitate the process by matching supply and demand, reducing information asymmetry, and improving the speed and quality of value co-creation. Through appropriate co-creation patterns and mechanisms aligned with the insights derived from the IUR network, the overall system efficiency will be improved.

### Management implications

This paper sheds light on the value co-creation in the IUR network of China’s high-end equipment manufacturing industry. Firstly, enterprises should establish a R&D investment supervision system to enhance capital utilization. A dedicated R&D investment management team comprising financial experts, R&D managers, and market analysts should be assembled. Particularly in high-end equipment manufacturing, which involves long-term and capital-intensive R&D projects, annual investment plans should be based on strategic goals and market trends, unlike the short-cycle consumer goods industries. Enterprises should then use advanced financial management software to track R&D funds and conduct monthly audits. Moreover, regular evaluations improve capital utilization, as R&D efficiency significantly impacts competitiveness in this industry. Key performance indicators such as R&D output per unit of investment and new product time-to-market should be established. In addition to supervising R&D investments, enterprises should enhance their capacity to absorb knowledge and establish a fair system for distributing innovation benefits within knowledge-intensive industries. Employee training is essential for cultivating a high-tech workforce and should include programs on emerging technologies, industry seminars, and international exchanges. Enterprises should also develop internal knowledge-sharing platform to manage patents and technical expertise. Furthermore, when designing a fair distribution system, factors such as the complexity of innovation, economic benefits, and team collaboration should be considered to enable a multidimensional evaluation of innovation contributions. Rewards may include bonuses, equity incentives, and opportunities for career advancement.

Secondly, academic institutions should establish formalized systems for assessing researchers’ achievements and implement innovation reward policies to incentivize their efforts, thereby enhancing overall R&D capabilities. Since high-end equipment R&D focuses on practical applications, unlike some basic research-oriented academic fields, assessment criteria should shift from traditional publication-based evaluations to metrics such as patent commercialization rates. Additionally, innovation reward policies should include significant prizes for breakthroughs in key high-end equipment technologies. Furthermore, joint research projects and workshops should be organized to facilitate knowledge exchange, which is crucial in high-end equipment manufacturing, where complex problems require interdisciplinary solutions. This approach enables academic institutions to collaborate more closely with industry partners, better understand their actual needs and adjust research directions accordingly.

Thirdly, the government should develop a more effective subsidy and penalty system. Subsidies out to be directed toward projects with high potential for value co-creation, while penalties should be calibrated to ensure effectiveness without discouraging participation. Specifically, incentive and regulatory policies tailored to the industry’s technological advancements should be implemented to ensure subsidies support projects that drive breakthroughs in key core technologies. Finally, it is essential to establish a robust science and technology intermediary service system that leverages market mechanisms alongside government guidance to promote value co-creation within the IUR network. Intermediaries should create specialized service platforms for high-end equipment technology transfer, train professionals who understand both technology and market demands, and encourage resource sharing among various types of intermediaries. They should enhance service quality by strengthening information management systems and expanding network connections. Additionally, they should offer more customized services to address the diverse needs of different agents within the IUR network.

### Limitations and future research

This paper examines the evolutionary dynamics and influencing factors of value co-creation within the IUR network from a multi-agent game perspective. It develops an evolutionary game model grounded in a realistic network, thereby enriching research in the field of IUR value co-creation. Despite its contributions, several limitations remain. First, the study focuses on four agents—enterprises, academic institutions, government, and intermediaries—to analyze the evolution of value co-creation within the IUR network, potentially overlooking other influential actors. Future research could incorporate additional perspectives, such as financial service institutions, and integrate them into the existing evolutionary game model. Second, the simulation analysis is based on the real IUR network in the high-end equipment manufacturing industry but does not account for the evolution of the network structure. Further investigation coupling the game model with dynamic network analysis is warranted. Third, the study uses China’s high-end equipment manufacturing industry as a case and conducts simulation analysis based on related patent data. The industry’s specificity and limitations of patent data may affect the generalizability of the findings. Future research could extend to other high-tech sectors, such as the semiconductor industry, and diversify data sources to include R&D project reports, industry news, and enterprise financial statements.

## Supporting information

S1 File The patent data was collected from the PATSNAP platform and supported the IUR network construction. (XLSX)

S1 Fig This shows the visualizations of additional simulation results. (PDF)
