# Supplementary figures and images for: Evolutionary dynamics of value co-creation within the industry-university-research network: A multi-agent game perspective
Source: PLoS One. 2025 Dec 4;20(12):e0338379. doi: 10.1371/journal.pone.0338379 (PMC12677796; doi:10.1371/journal.pone.0338379)

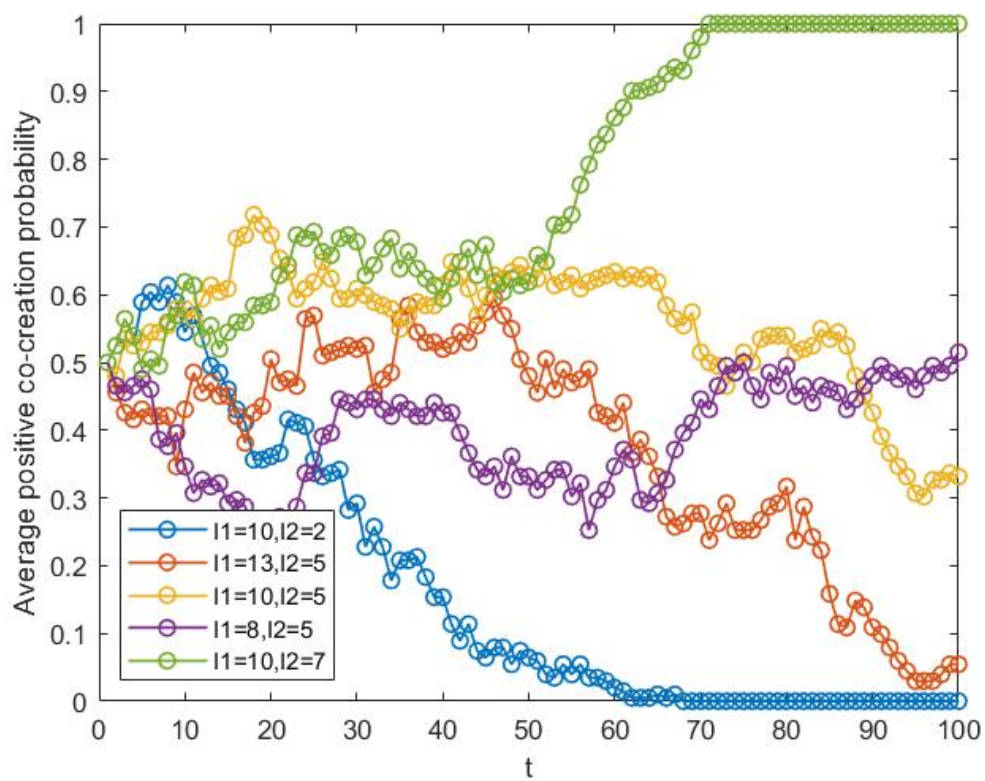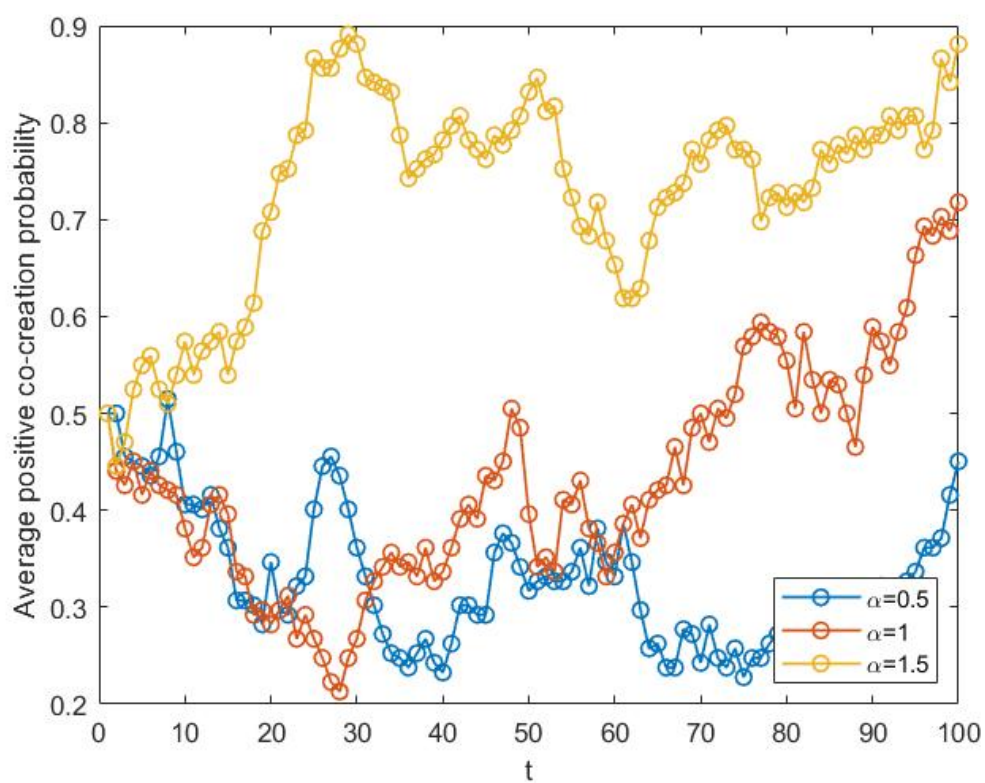

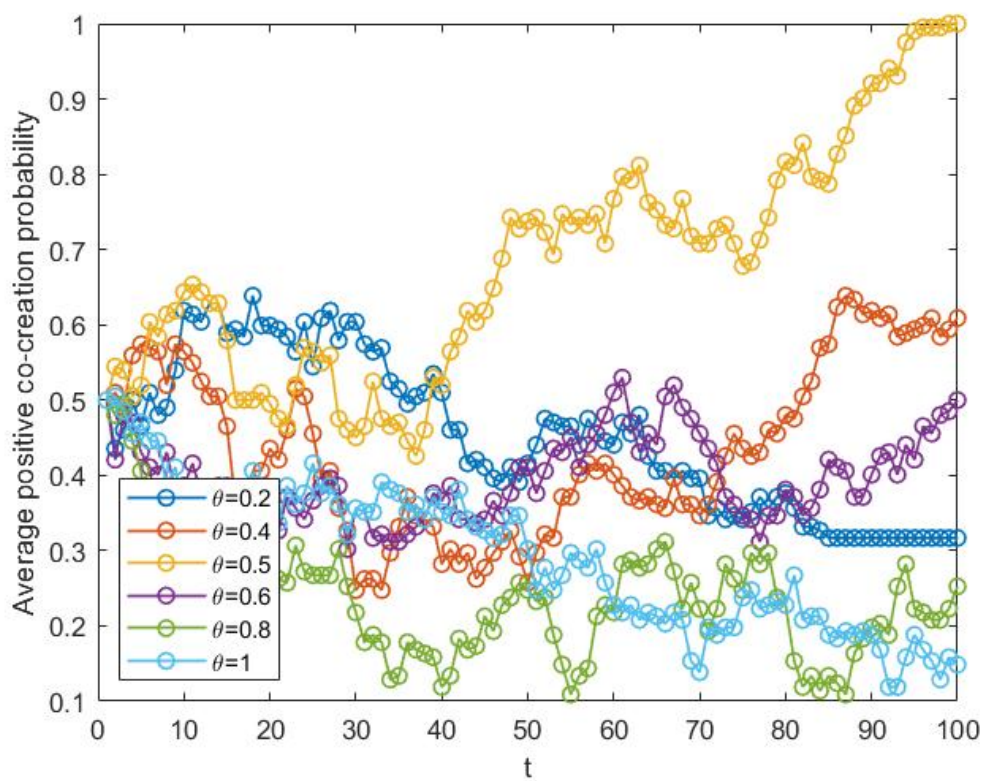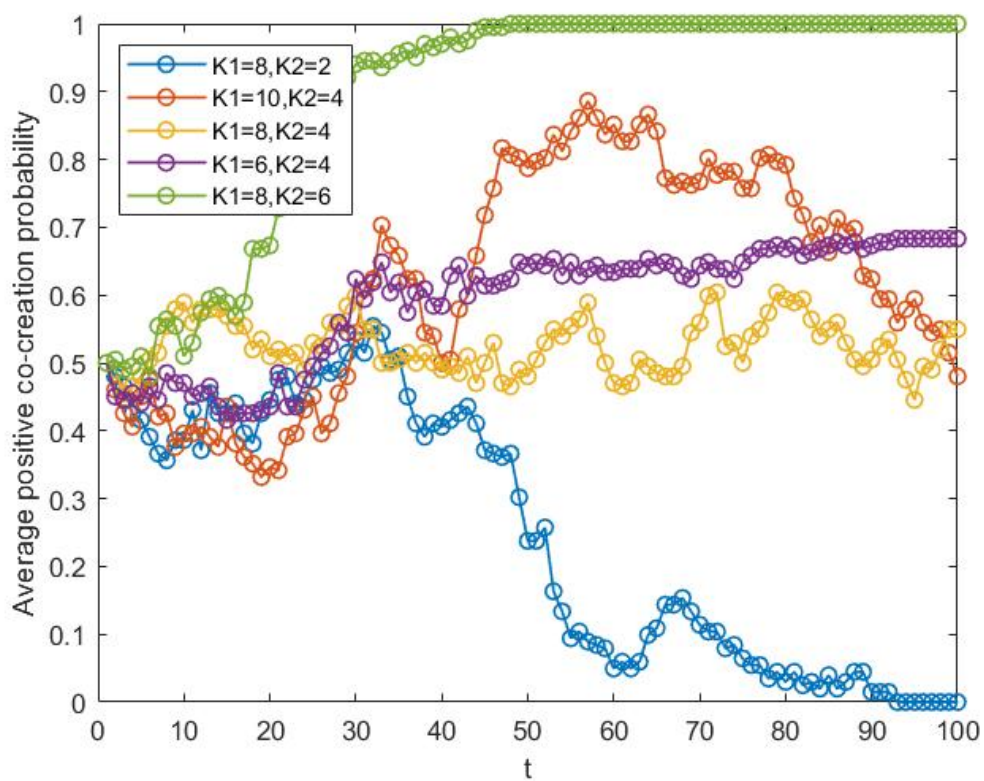

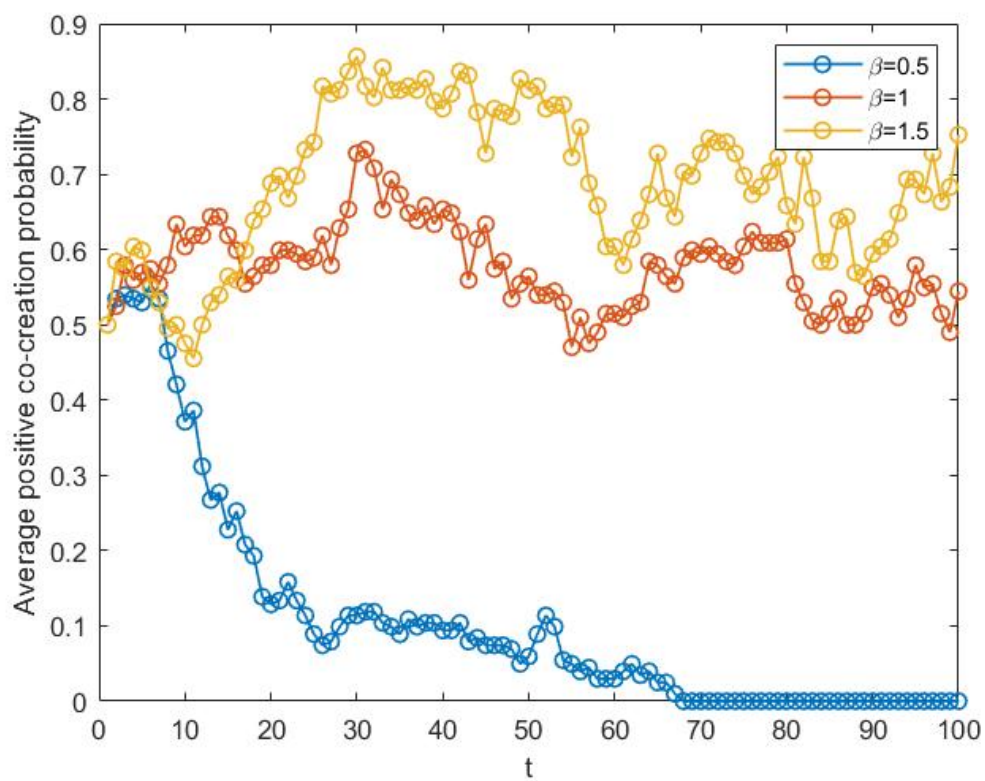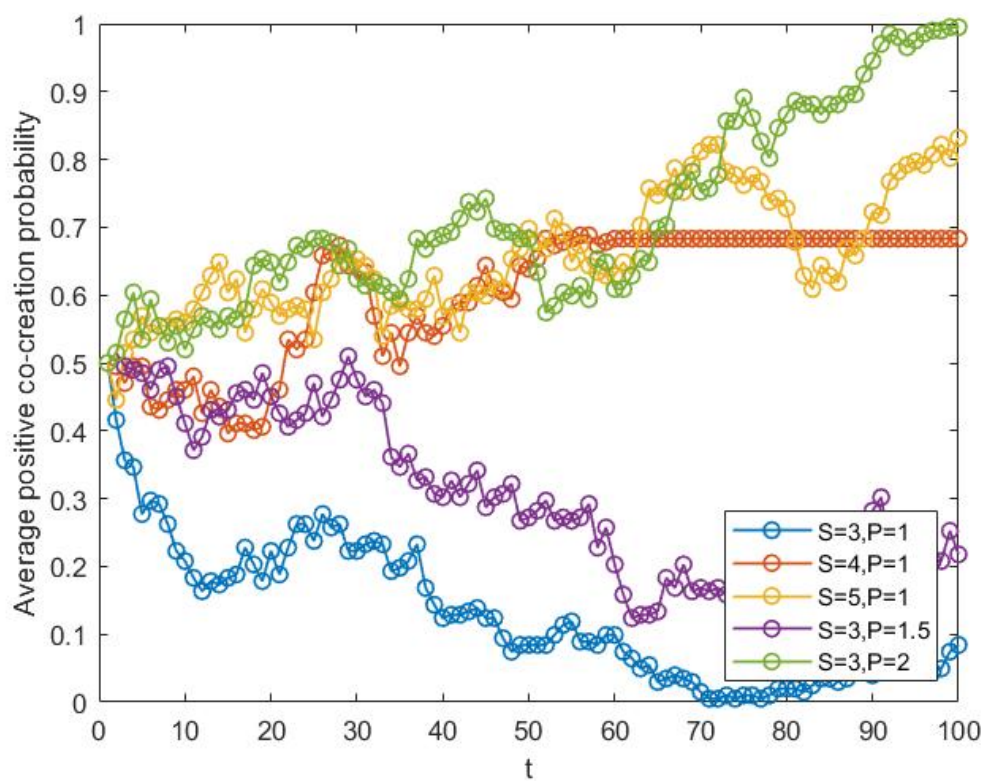

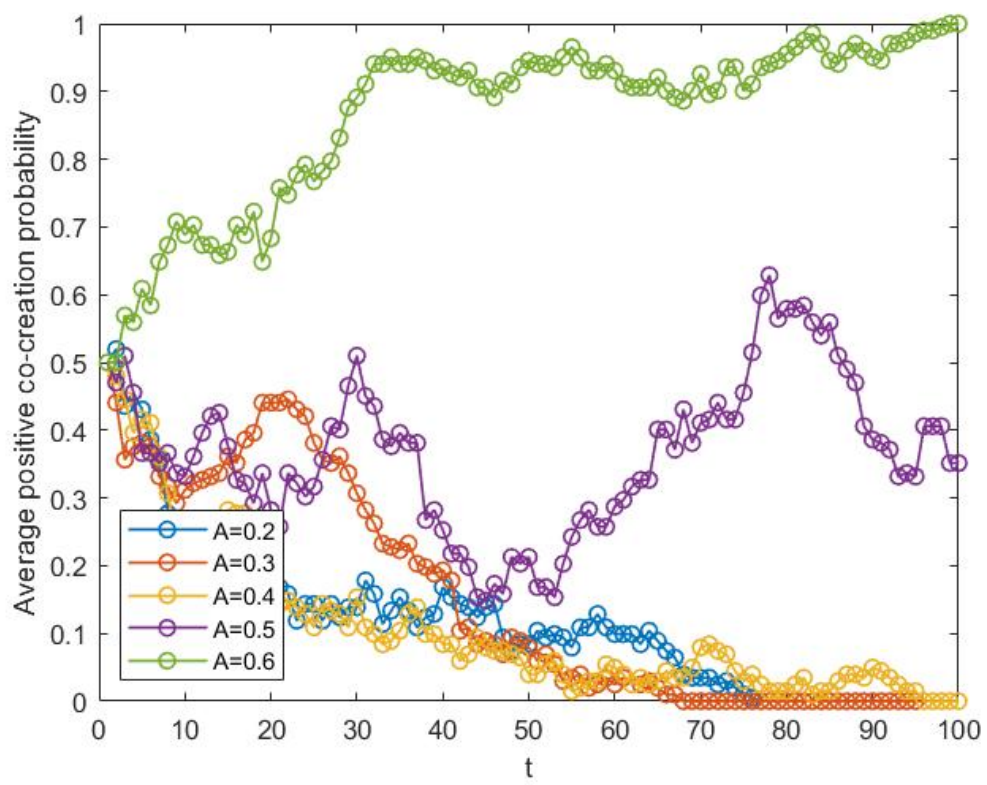

Supplement: S1 Fig — (PDF) [file pone.0338379.s002.pdf]
